# Thermal acclimatisation to heatwave conditions is rapid but sex-specific in wild zebra finches

**DOI:** 10.1038/s41598-023-45291-0

**Published:** 2023-10-25

**Authors:** Anaïs Pessato, Eve Udino, Andrew E. McKechnie, Andrew T. D. Bennett, Mylene M. Mariette

**Affiliations:** 1 Centre for Integrative Ecology, School of Life and Environmental Sciences, Deakin University, Geelong 3216, VIC Australia; 2 South African Research Chair in Conservation Physiology, South African National Biodiversity Institute, Pretoria, 0001 South Africa; 3 DSI-NRF Centre of Excellence at the FitzPatrick Institute, Department of Zoology and Entomology, University of Pretoria, Pretoria, 0001 South Africa; 4 One Health Research Group, Melbourne Veterinary School, Faculty of Science, University of Melbourne, Werribee, VIC 3030 Australia; 5 Doñana Biological Station EBD-CSIC, 41092 Seville, Spain

**Keywords:** Metabolism, Ecology, Behavioural ecology, Climate-change ecology, Conservation biology, Ecophysiology, Evolutionary ecology

## Abstract

Under climate change, increasing air temperature average and variability pose substantial thermal challenges to animals. While plasticity in thermoregulatory traits could potentially attenuate this impact, whether thermal acclimatisation can occur quickly enough to track weather variability in hot climates is unknown in any endotherm, and sex differences have never been tested. We investigated acclimatisation responsiveness of male and female wild zebra finches to short-term (< 2 weeks) summer temperature fluctuations in the Australian desert. Hotter weather before respirometry trials triggered a typical acclimatisation response (especially at chamber temperature T_chamb_ ≥ 40). However, acclimatisation occurred remarkably rapidly: metabolic rate responded within just one day, while body temperature (T_b_) and evaporative cooling capacity (EHL/MHP) were best predicted by weather on the trial day; whereas evaporative water loss responded more slowly (1 week). Nonetheless, rapid acclimatisation only occurred in males, and females had higher T_b_ and lower EHL/MHP than males, potentially increasing hyperthermia risk. Furthermore, acclimatisation did not translate into greater acute heat tolerance (i.e. ability to tolerate T_chamb_ = 46 °C). Our results therefore reveal surprisingly rapid acclimatisation and even anticipatory adjustments to heat. However, with no changes in acute heat tolerance, and in females, phenotypic flexibility may provide only limited buffering against the detrimental impact of heatwaves.

## Introduction

As a consequence of anthropogenic climate change, global surface temperature and the frequency of heatwaves are increasing dramatically^1^. In many regions, these changes have already resulted in loss of biodiversity^2^. Mass die-offs during heatwaves have been documented across diverse taxa^3–5^, presumably because conditions exceeded individuals’ thermoregulatory limits. Indeed, based on species’ physiological limits, widespread and severe population declines are predicted under future climates, through effects on survival and reproduction^6–8^.

Nonetheless, phenotypic plasticity or “phenotypic flexibility” (*sensu*^9^) in traits related to heat tolerance—whereby thermoregulatory performance is reversibly adjusted to prevailing conditions through acclimatisation – may have the potential to partly buffer the negative impacts of climate change^10,11^. If so, incorporating phenotypic flexibility in physiological traits into predictive models of species distributions may yield more accurate predictions of species vulnerability to climate change^12,13^. Crucially, however, such beneficial effects of physiological flexibility rely on thermal physiology tracking weather variability over time. While phenotypic flexibility can lessen the physiological cost of seasonal climate variation, such responses may become maladaptive when the weather varies faster than a species’ acclimatisation capacities^14^. By contrast, if acclimatisation responses are rapid, organisms may be able to anticipate and prepare for upcoming weather by adjusting their physiology to recent or current conditions.

Acclimatisation to weather conditions, or acclimation to experimentally-manipulated thermal environments, are well-studied physiological responses occurring in many endotherms^13,15,16^. However, it is generally assumed that acclimation takes at least 2 weeks to arise, and experiments therefore typically use acclimation periods of 2–4 weeks, without investigating the effects of acclimation period duration^15,17^. Only a handful of studies have measured the time-course of acclimation or acclimatisation among endotherms. To the best of our knowledge, only two studies (5 species in total) have measured avian acclimation or acclimatisation over < 7 days^18,19^. These authors found that, with the notable exception of the American tree sparrow (*Spizella arborea*; tested in both studies), acclimation to constant temperature in captivity does not occur within 8 days, and the weather in the past 14–30 days better explains variations in winter resting metabolic rate in free-living birds than weather on shorter timescales^18,19^. In rodents nonetheless, while acclimation took 2–5 weeks under constant temperature to reach a maximal level, all three species showed noticeable response within the first week of temperature change^20–22^. Overall, the evidence for acclimation timing is therefore very scarce and species-dependent. In addition, all avian or mammalian studies to date have exclusively focussed on responses to cold or mild conditions. With no data on the rates of thermoregulatory adjustments to hot conditions (maximum temperature tested = 30 °C), the functional significance of acclimatisation in alleviating or worsening the impact of increasing heatwaves on populations remains unclear. Furthermore, unlike for thermoregulation efficiency, acclimatisation of heat-tolerance limit in birds has received little attention^23,24^. Nonetheless, in white-browed sparrow-weavers (*Plocepasser mahali*), heat tolerance, measured as the air temperature at which severe hyperthermia was reached (i.e. body temperature ≥ 44.5 °C), was higher in summer than winter in one arid population (but not in two mesic populations)^23^, and in individuals acclimatised for 30 days to 42 °C compared to those kept at 30 °C or 36 °C^24^. Both studies together suggest that acclimatisation of heat tolerance may occur under very high temperatures, but this remains to be tested in other systems.

Hot deserts at subtropical latitudes are some of the most rapidly warming regions on the planet^25^, and are characterised by highly variable summer air temperatures and unpredictable precipitation^26,27^. Species inhabiting hot arid habitats are thus thought to be highly vulnerable to climate change, but they may also potentially be more physiologically plastic, if more variable climate (e.g. at higher latitudes) selects for greater flexibility. Whether this “climatic variability hypothesis”^23,28^ (but see^29^) extends to more rapid acclimatisation to short-term weather variability has not be tested. Here, we investigated the timing of acclimatisation to summer weather conditions in free-living individuals of a desert specialist, the Australian zebra finch (*Taeniopygia castanotis*). We quantified rates of acclimatisation in both males and females, as thermoregulation capacities in the heat may differ between the sexes^30^, and females have been reported to have higher T_b_ than males in wild-derived captive zebra finches^30^ and several other avian species^31^. In contrast to cold or thermoneutral conditions^31,32^, sexual dimorphism in thermoregulatory performance in the heat has rarely been tested, with studies finding mixed results^24,30,33,34^. Yet, evaluating sex differences in thermoregulation is essential, given the negative impact of extreme heat on reproduction^35^ and the consequences this may have on population persistence under climate change^36,37^.

**Figure 1:**
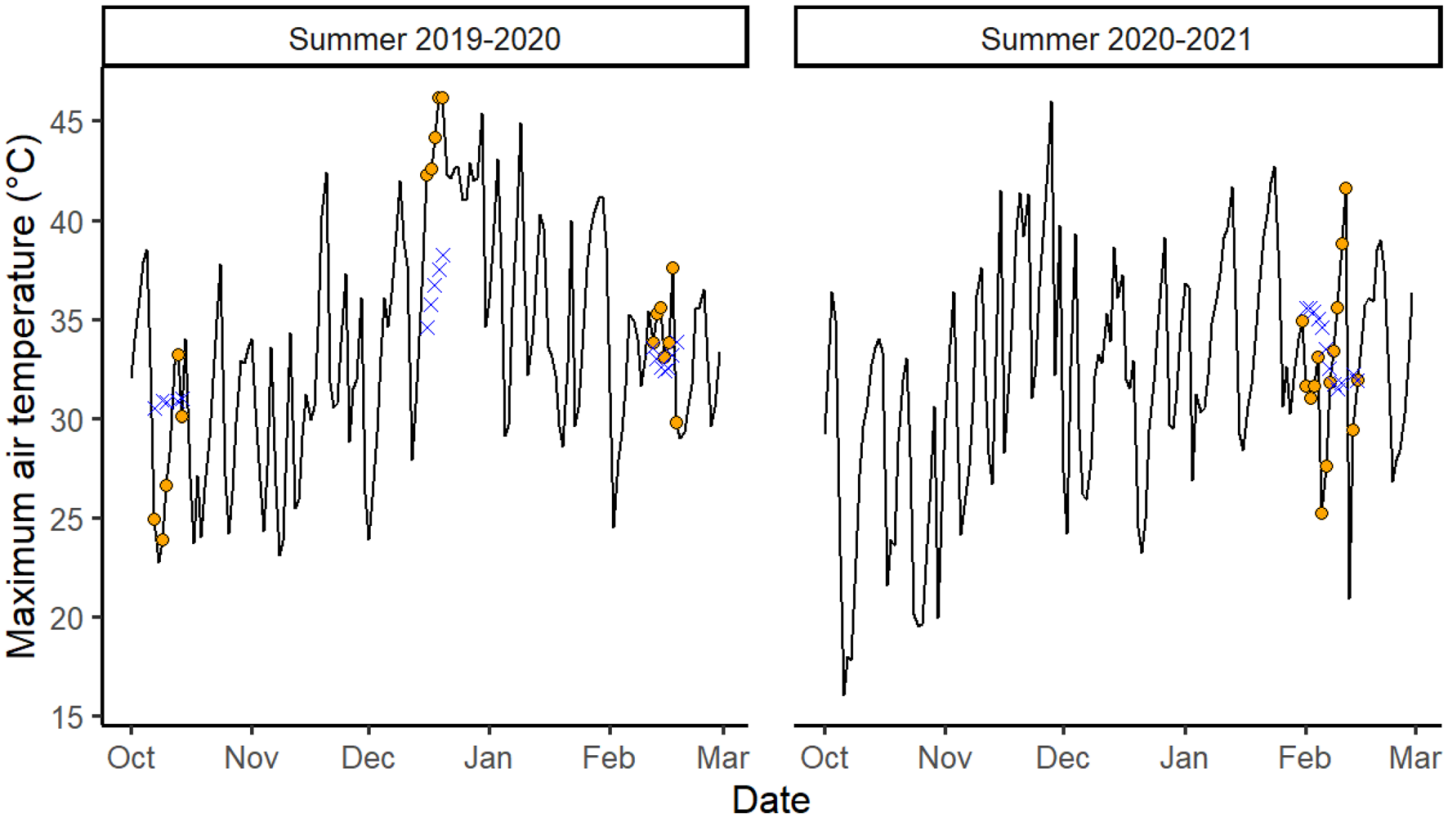
Daily maximum air temperature (black line) during summers 2019–2020 and 2020–2021 on the days of respirometry measurements (T_0day_; orange points), and averaged over the 2 weeks prior (T_−2weeks_; blue crosses), recorded at Leigh Creek Airport. No field trip could occur during Oct 2020–Jan 2021 because of Covid-19-related border closures.

We used an open flow-through respirometry system to measure metabolic rate (MR), evaporative water loss (EWL) and body temperature (T_b_) of individuals exposed to air temperatures (T_a-chamb_) ramping gradually from 31 °C up to 46 °C. To characterize the timing of acclimatisation to natural weather fluctuations (Fig. 1), we considered the maximum air temperature (T_a_) at different time scales (i.e. on the day of the experiment (T_0day_), the day before (T_−1day_), or over the preceding 3 days (T_−3days_), 1 week (T_−1week_) and 2 weeks (T_−2weeks_)), and then tested which time interval best explained the observed variation in thermoregulatory values. Similarly to changes observed in summer-acclimatised or heat-acclimated individuals across avian species^15,23,38–40^, we predicted that exposure to heatwave conditions before measurement would be associated with lower MR and higher evaporative cooling capacity (EHL/MHP), whereas EWL may be lower (to conserve water). We also expected these changes to be associated with lower T_b_ and greater acute heat tolerance after hot weather. In addition, we predicted that zebra finches, as desert specialists, may acclimatise rapidly in response to changing weather conditions, except, potentially, when temperature deviations from the day before are too large (measured as the difference in temperatures between T_0day_ and T_−1day_ (ΔT_0–1_), or T_−1day_ and T_−2days_ (ΔT_1–2_)). Lastly, we predicted that free-ranging females may have higher T_b_ than males^31^, and that the sexes may differ in evaporative cooling capacity^30^, and, potentially, the timing of acclimatisation.

## Results

### Rapid acclimatisation in thermoregulatory traits

**Figure 2:**
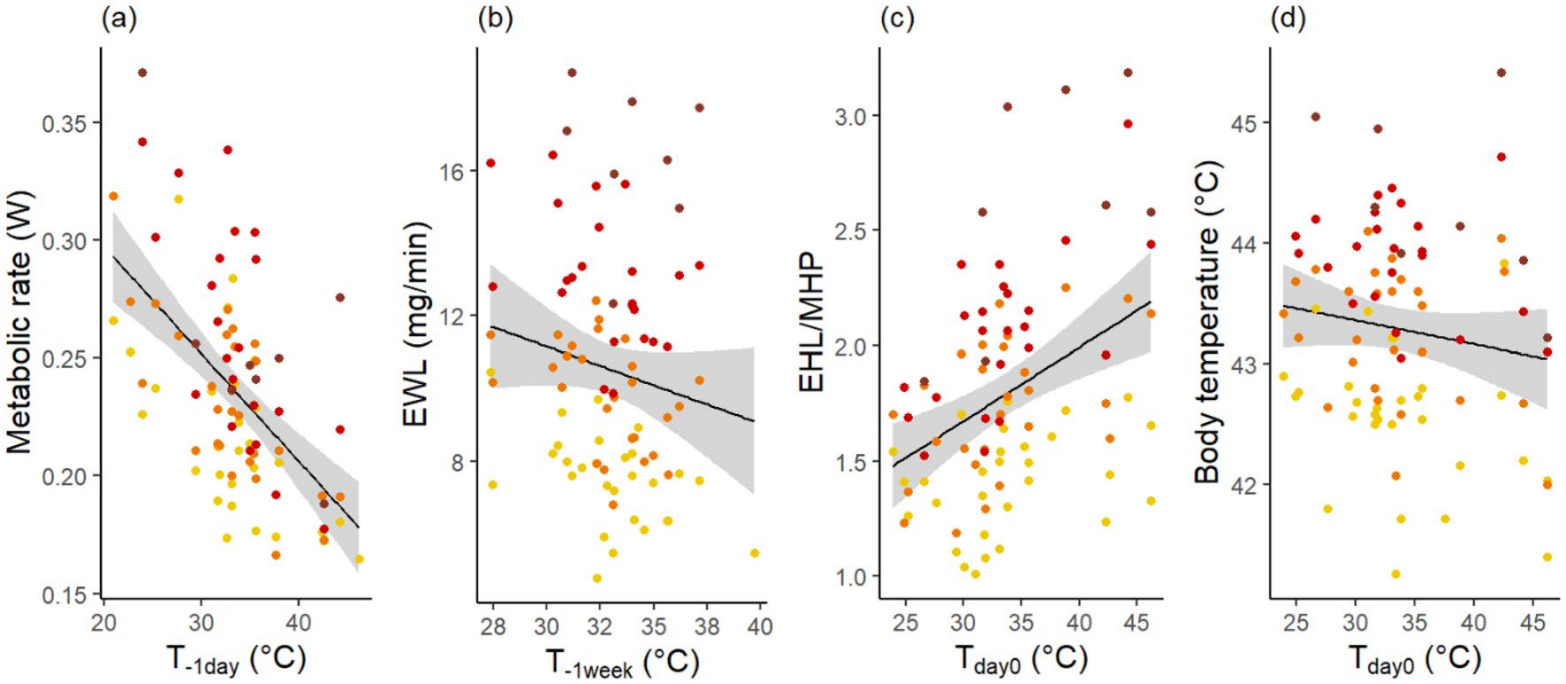
Effects of weather predictors on thermoregulation at T_a-chamb_ ≥ T_uc_ (38 °C). Effects of (**a**) the maximum air temperature the day before measurement (T_−1day_) on metabolic rate; (**b**) the average maximum air temperature 1 week prior to measurement (T_−1week_) on evaporative water loss (EWL); and (**c**,**d**) the maximum air temperature on the day of measurement (T_0day_) on (**c**) EHL/MHP and (**d**) body temperature. Colours corresponds to T_a-chamb_ (yellow = 40 °C, orange = 42 °C, red = 44 °C and brick = 46 °C). Regression lines and confidence intervals (grey area) display significant effects from model-averaging. Excluding the point at T_−1week_ = 39.7 °C for EWL did not affect significance.

All thermoregulatory traits responded to weather conditions (measured as air temperature) prior to respirometry trials, at least at high chamber temperatures (i.e. T_a-chamb_ ≥ 40; above the upper critical limit of thermoneutrality T_uc_), as well as below the T_uc_ for body temperature T_b_ (i.e. null/base models [without weather predictors] not retained in best model sets, Table 1A). As expected, after hot weather, metabolic rate (MR) and T_b_ were significantly lower, and evaporative cooling capacity (EHL/MHP) significantly higher (CIs excluded zero, Table 1B, Fig. 2). These changes occurred surprisingly rapidly, with the air temperature the day preceding the trial (T_−1day_) explaining thermoregulatory values better (i.e. T_−1day_ in best model sets) than longer-term weather predictors for all traits except EWL (Table 1A). Notably, T_−1day_ was 2.3 to 9.8 AICc units lower than the typical acclimatisation period of 2 weeks. Remarkably, even the maximum air temperature on the day of measurement (T_0day_) influenced T_b_ and EHL/MHP (Table 1, Fig. 2), indicating that individuals were prepared for upcoming conditions, probably by responding to morning temperatures at time of capture (Pearson correlation between T_0day_ (i.e. maximum temperature) and the morning temperature at time of capture: r = 0.98 p < 0.001). For all three traits, adding the amplitude of temperature deviation from the day before (ΔT) did not improve models, suggesting that large sudden weather changes did not impede rapid acclimatisation. These effects of T_−1day_ and T_0day_ were significant at T_a-chamb_ both below and above the T_uc_, although weaker in the former (base/null model retained < T_uc_ for MR and EHL/MHP; Table 1). By contrast to other traits, evaporative water loss adjusted more slowly: EWL at moderate T_a-chamb_ (< T_uc_) did not vary with weather, and EWL at high T_a-chamb_ (> = 40 °C) only adjusted within a week, decreasing following a hot week (Table 1, Fig. 2). When removing seasonal variation by restricting analyses to February trips, results were qualitatively unchanged, with short- to medium-term weather (T_−1day_, T_0day_, T_−1week_) explaining significant variations in MR, EHL/MHP and EWL (respectively) at high T_a-chamb_, whereas longer-term weather (T_−2week_) did not. Effects for T_b_ were however no longer detected, possibly because the sample size was reduced by half (Table S4).

**Table 1:** (A) Top model set (ΔAICc ≤ 2), and (B) model-averaged estimates of predictors included in the top models, for metabolic rate, evaporative water loss, evaporative cooling capacity and body temperature, below and above T_uc_ on the full dataset (n = 29 birds).

Metabolic rate	T_a-chamb_ < T_uc_					T_a-chamb_ > T_uc_				
(A) Model	df	LL	AICc	ΔAICc	w	df	LL	AICc	ΔAICc	w
Null	3	103.7	− 201.0	1.1	0.268					
+ T− _1day_	4	105.4	− 202.1	0	0.470	5	179.3	− 347.9	0	0.839

Metabolic rate	T_a-chamb_ < T_uc_					T_a-chamb_ > T_uc_				
(B) Predictors	Est	SE	CI			Est	SE	CI		
Intercept	0.218	0.007	**0.21; 0.23**			0.237	0.005	**0.23; 0.25**		
T_a-chamb_						0.042	0.005	**0.03; 0.05**		
T_−1day_	− 0.043	0.012	− **0.07; **− **0.02**			− 0.053	0.009	− **0.07; **− **0.03**		

Evaporative water loss	T_a-chamb_ < T_uc_					T_a-chamb_ > T_uc_				
(A) Model	df	LL	AICc	ΔAICc	w	df	LL	AICc	ΔAICc	w
Base	4	− 79.2	167.3	0	0.280					
+ T_−1week_ + sex						7	− 131.8	279.0	0	0.373

Evaporative water loss	T_a-chamb_ < T_uc_					T_a-chamb_ > T_uc_				
(B) Predictors	Est	SE	CI			Est	SE	*CI*		
Intercept	3.465	0.151	**3.17; 3.76**			10.567	0.197	**10.20; 10.94**		
Mass	0.788	0.305	**0.19; 1.38**			1.491	0.378	**0.78; 2.20**		
T_a-chamb_						5.624	0.199	**5.22; 6.01**		
T_−1week_						− 1.049	0.389	− **1.78; **− **0.32**		
Sex						0.892	0.395	**0.15; 1.64**		

Evaporative cooling capacity	T_a-chamb_ < T_uc_					T_a-chamb_ > T_uc_				
(A) Model	df	LL	AICc	ΔAICc	w	df	LL	AICc	ΔAICc	w
Null	3	20.1	− 33.8	0	0.409					
+ T_−1day_	4	20.7	− 32.6	1.2	0.223	5	2.0	6.8	1.0	0.153
+ T_0day_	4	20.3	− 31.9	2	0.152	5	2.4	5.9	0	0.247
+ T_0day_ + sex						6	3.4	6.3	0.5	0.195
+ T_−1day_ + sex						6	3.2	6.6	0.7	0.171

Evaporative cooling capacity	T_a-chamb_ < T_uc_					T_a-chamb_ > T_uc_				
(B) Predictors	Est	SE	CI			Est	SE	CI		
Intercept	0.642	0.021	**0.60; 0.68**			1.781	0.044	**1.69; 1.87**		
T_a-chamb_						0.657	0.038	**0.58; 0.73**		
T_0day_	0.094	0.042	**0.01; 0.18**			0.278	0.087	**0.11; 0.45**		
T_−1day_	0.100	0.042	**0.02; 0.18**			0.263	0.085	**0.09; 0.43**		
sex						0.199	0.084	**0.03; 0.37**		

Body temperature	T_a-chamb_ < T_uc_					T_a-chamb_ > T_uc_				
(A) Model	df	LL	AICc	ΔAICc	w	df	LL	AICc	ΔAICc	w
+ T_0day_ + sex	6	− 43.4	100.4	0	0.583	6	− 36.2	85.4	0	0.225
+ Sex						5	− 37.4	85.6	0.2	0.208
+ T_−1day_ + sex						6	− 36.4	85.8	0.4	0.188

Body temperature	T_a-chamb_ < T_uc_					T_a-chamb_ > T_uc_				
(B) Predictors	Est	SE	CI			Est	SE	CI		
Intercept	41.380	0.103	**41.18; 41.58**			43.305	0.086	**43.13; 43.48**		
T_a-chamb_	0.052	0.016	**0.02; 0.08**			1.329	0.055	**1.22; 1.44**		
T_0day_	− 0.623	0.205	− **1.01; **− **0.23**			− 0.351	0.168	− **0.69; **− **0.02**		
T_−1day_						− 0.328	0.164	− **0.65; < **− **0.01**		
Sex	− 0.688	0.208	− **1.09; **− **0.29**			− 0.521	0.173	− **0.86; **− **0.18**		

A predictor has a significant effect (bold) if CI excludes 0. Female is the reference group for sex. *df*’: degree of freedom, *LL:* log-likelihood, *ΔAICc*: difference in AICc scores between the best model and the model being compared, *w:* model weight, *Est:* parameter estimate, *SE:* standard error, *CI*: 95% confidence interval.

### Sexual dimorphism in thermoregulatory capacities

The timing of acclimatisation differed between the sexes. At high T_a-chamb_ (> T_uc_), only males adjusted all thermoregulatory traits to short-term weather. Males’ evaporative cooling capacity, T_b_ and MR showed the same patterns as in the pooled dataset, responding to T_0day_ and T_−1day_ (although, for EHL/MHP, the base model was included in the best model set: ΔAICc with T_0day_ = 1.5, Table S7A). By contrast, females’ evaporative cooling capacity (EHL/MHP) and T_b_ did not adjust to weather, and their MR response was more ambiguous and potentially slower than males’ (T_−1day_, T_−3days_, and T_−1week_ all significant, Table S7). Furthermore, EWL in males responded to weather 1–3 days before measurements at high T_a-chamb_, whereas there were no significant weather predictors for EWL in females. At T_a-chamb_ < T_uc_, sex differences were less consistent and weaker (null model included in best model sets, except for females’ T_b_): only males adjusted EHL/MHP (as for > T_uc_), but only females adjusted T_b_ and MR to recent weather. EWL below the T_uc_ was not significantly affected by weather in either sex, similar to the pooled dataset (Table S7).

**Figure 3:**
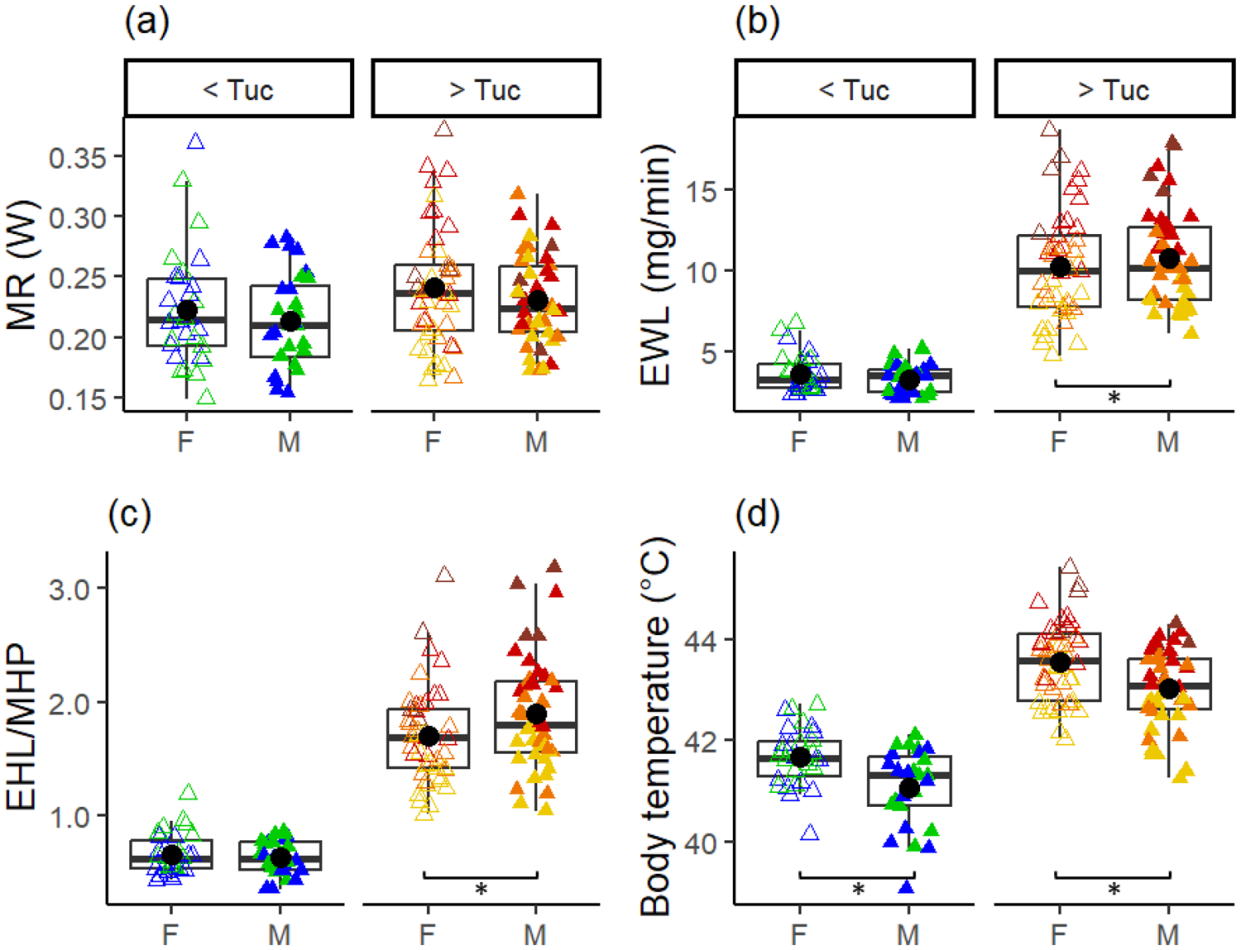
Sex differences in (**a**) metabolic rate (MR), (**b**) evaporative water loss (EWL), (**c**) evaporative cooling capacity (EHL/MHP) and (**d**) body temperature, at mild (left) and high (right) chamber temperatures, (i.e. T_a-chamb_ below or above T_uc_ = 38 °C respectively). Black dots show the mean in females (open triangles) and males (filled triangles). Colours corresponds to T_a-chamb_ (blue = 30 °C, green = 35 °C, yellow = 40 °C, orange = 42 °C, red = 44 °C and brick = 46 °C). * indicates a significant sex effect (i.e. CI excludes zero). Body temperature as a function of T_a-chamb_ is shown in Fig. S2.

Consistent with these sex differences in acclimatisation timing, females had overall higher T_b_ than males, both below and above the T_uc_ (i.e. sex significant in the pooled dataset: Table 1B, Fig. S2), and lower EWL and EHL/MHP than males at T_a-chamb_ > T_uc_ (Table 1, Fig. 3). By contrast, MR did not differ between the sexes (Table 1, Fig. 3). Taken together, these results reveal that females had lower and less flexible heat dissipation capacity than males at high air temperature, associated with a higher T_b_ at high but also mild T_a-chamb_.

### No acclimatisation or sex differences in acute heat tolerance

In spite of thermoregulatory traits rapidly adjusting to weather variations (i.e. air temperatures), no clear benefits for acute heat tolerance were found. Indeed, none of the weather predictors had a significant effect on either the probability of reaching T_a-chamb_ = 46 °C or trial completion probability (15 min at T_a-chamb_ = 46 °C), although for both proxies, some weather predictors explained some variation (i.e. retained in overall dataset model set: Table 2, and in February dataset: Table S5). The weakness of weather effects may be partly related to the fact that, in contrast to results for thermoregulatory traits, sudden changes from previous conditions (ΔT) appeared to have some influence on heat tolerance (i.e. retained in the top model set, Table 2A), albeit not significantly (Table 2B). In agreement, heat tolerance proxies did not improve after heatwaves in either sex, when the sexes were considered separately (Table S8). Finally, males and females achieved similar heat tolerance thresholds (i.e. sex not retained in top models for either heat tolerance proxy, Table 2A), even though their evaporative cooling capacity differed.

**Table 2:** (A) Top model set (ΔAICc ≤ 2 from best model), and (B) model-averaged estimates of predictors included in the top models for T_a-max_ (46 °C or less) and trial completion (i.e. stayed 15 min at T_a-chamb_ = 46 °C).

Models	T_a-max_ = 46 °C (yes/no)					Trial completion				
	df	LL	AICc	ΔAICc	w	df	LL	AICc	ΔAICc	w
(A)										
Null	**1**	− **20.2**	**42.5**	**0.5**	**0.152**	**1**	− **20.2**	**42.5**	**1.3**	**0.072**
Base	**2**	− **18.8**	**42.0**	**0**	**0.191**	**2**	− **18.6**	**41.7**	**0.5**	**0.105**
+ ΔT_0–1_ + T_0day_	**4**	− **16.7**	**43.0**	**1.0**	**0.115**					
+ T_−2weeks_	**3**	− **18.4**	**43.7**	**1.7**	**0.081**					
+ T_0day_						**3**	− **17.1**	**41.2**	**0**	**0.138**
+ T_−1day_						**3**	− **17.2**	**41.4**	**0.2**	**0.125**
+ T_−1week_						**3**	− **17.3**	**41.4**	**0.2**	**0.123**
+ ΔT_1–2_ + T_−1day_						**4**	− **16.4**	**42.4**	**1.2**	**0.075**
+ T_−3days_						**3**	− **17.8**	**42.4**	**1.3**	**0.073**

Predictors	T_a-max_ = 46 °C (yes/no)					Trial completion				
	Est	SE	CI			Est	SE	CI		
(B)										
Intercept	0.660	0.405	− 0.17; 1.49			− 0.758	0.446	− 1.67; 0.15		
Capt. time	1.587	0.963	− 0.38; 3.55			1.764	1.002	− 0.28; 3.81		
T_0day_	0.189	0.915	− 1.69; 2.07			1.505	0.915	− 0.28; 0.81		
ΔT_0–1_	− 2.039	1.248	− 4.60; 0.52							
T_−1day_						1.457	1.046	− 0.68; 3.60		
ΔT_1–2_						1.887	1.606	− 1.41; 5.18		
T_−3days_						1.195	0.945	− 0.74; 3.13		
T_−1week_						1.624	1.062	− 0.55; 3.80		
T_−2weeks_	− 0.702	0.836	− 2.41; 1.01							

A predictor has a significant effect (bold) if CI excludes 0. Abbreviations as in Table 1.

## Discussion

Our study demonstrates that zebra finches adjust thermoregulatory performance in response to prevailing weather, but sexes differ in such phenotypic flexibility. Remarkably, adjustments in metabolic rate, evaporative cooling capacity and body temperature occurred very rapidly—within a day—to summer air temperature fluctuations, whereas evaporative water loss changed within 1 week (Fig. 2). Interestingly, only males rapidly adjusted all four thermoregulatory traits at high T_a-chamb_, with MR, EWL and T_b_ decreasing and EHP/MHP increasing, following or during a hot day. Accordingly, males had lower T_b_ at all T_a-chamb_ and higher EHL/MHP at high T_a-chamb_ than females, although their acute heat tolerance remained similar to that of females. Overall, our study reveals very rapid sex-specific acclimatisation to heatwave conditions in a desert passerine, but without changes to acute heat tolerance. These findings highlight the need to thoroughly evaluate acclimatisation capacities across species, so the role of phenotypic plasticity in responding to climate change can be elucidated.

Metabolic rate, body temperature and evaporative cooling capacity adjusted surprisingly rapidly to weather conditions on the day before, or even on the day of measurements. Importantly, these changes were not driven by longer-term or seasonal effects, since weather in the preceding 2 weeks had no effect, and results were maintained when only February data were considered. Our results are contradictory to the notion of acclimatisation occurring over time scales of weeks in endotherms, and, at first sight, appear to contrast with previous findings on rodents demonstrating that under constant T_a_, complete acclimation takes several weeks to establish^20–22^. However, in nature, environmental temperatures are very rarely constant, as they usually vary both within and between days. Whether the maximum acclimation levels measured in the lab are actually attainable under natural fluctuating conditions is therefore questionable. Remarkably however, at least in wild zebra finches, the magnitude of the changes we found is similar to those typically observed after 3–4 weeks of acclimation^39,40^. Indeed, in our study, MR decreased by 39%, and EHL/MHP increased by 57%, over T_−1day_ values increasing from 21 to 46 °C (Fig. 3). Such decrease in MR (1.5% MR.°C^−1^) is similar to that measured after 2–4 weeks of acclimation in captive zebra finches^39,41^, or other wild-caught passerines^17,40^. Interestingly, the rapid thermoregulatory adjustments we document were not negatively impacted by sudden changes in thermal conditions (ΔT). Taken together, our findings support our hypothesis that the capacity for rapid physiological acclimatisation may have adaptive value in some habitats, such as arid unpredictable habitats (as in the Australian desert: Fig. 1)^26,27^. Further studies on a range of species and environments are nonetheless needed to determine whether acclimatisation is more rapid among species inhabiting desert environments, and to measure the fitness impact of such acclimatisation. Nevertheless, rapid acclimation may be more widespread than currently acknowledged, given that, in the temperate zone, American tree sparrows also acclimate very rapidly^18,19^, and that in great tits (*Parus major*) short-term (i.e. past week) weather explained variation in MR whereas that experienced previously (i.e. in the fortnight from week 2 to 4 before testing) did not^42^.

We also found that EWL responded more slowly than MR at high T_a-chamb_, and phenotypic flexibility in EWL was not evident at T_a-chamb_ < T_a_. This is consistent with the literature, where the direction of change in EWL with acclimation temperature is less consistent across studies than for MR, and effects can differ below and above the T_uc_^23,38,40^. This might be because of the underlying mechanisms allowing MR versus EWL flexibility (e.g. changes in muscle versus skin ultrastructure^43–45^) and/or because cutaneous (principal component of EWL < T_uc_) and respiratory EWL (occurring mostly > T_uc_) may respond differently to acclimation^39^. Furthermore, other factors such as developmental plasticity, have been shown to contribute to variation in EWL, including at adulthood in the zebra finch^30^. Lastly, beyond temperature, EWL may acclimate to humidity, as for example, in house sparrow (*Passer domesticus indicus*) fledglings acclimated to dry conditions^46^ or nestlings acclimatised to desert environments^45^. More studies are clearly needed on the drivers and time course of EWL acclimatisation, including in a range of habitats where selection strength by lethal dehydration may differ.

Despite efficiently adjusting their thermoregulation capacities to heatwave conditions, zebra finches’ tolerance of acute heat exposure under experimental conditions did not improve. Instead, sudden weather changes might have disrupted acclimatisation of heat tolerance, although more data is needed to confirm this non-significant effect. These results are nonetheless consistent with the interpretation that temperatures at our study site may not have been high or stable enough for heat tolerance to improve, given white-browed sparrow-weavers increased heat-tolerance only at very high acclimation or acclimatisation temperatures^23,24^. Indeed, it is possible that flexibility of traits other than thermoregulation capacity that contributes to individual thermal limits (e.g. heat-shock protein regulation, mitochondrial efficiency^47^), may have restricted heat tolerance adjustments. In addition, we found that heat tolerance did not vary between the sexes, even though thermoregulation differed, as also found by^33^.

To our knowledge, our study provides the first evidence among endotherms for sex-specific thermal acclimatisation, with male zebra finches acclimatising rapidly whereas females showed no response. This is particularly interesting considering that zebra finches have no sexual dimorphism in body-size or microsite use^48^ and little behavioural differences^49^. By contrast, in ectotherms, a recent meta-analysis found that on average females show greater heat tolerance plasticity than males (but only among free-living animals^50^). In addition, we showed that female zebra finches have higher T_b_ and lower EHL/MHP than males, consistent with previous findings in captive wild-derived individuals^30^ and, in the case of T_b_, other avian species^31,34^. Most previous studies have found either no sex dimorphism in T_b_^24,33^ or higher T_b_ in females^31,34^, but there is to date no clear explanation for female higher T_b_, or even for the presence or absence of sexual dimorphism in thermoregulatory traits across species. Nonetheless, implications of such sexes differences for acute heat tolerance are unclear, since we found no sexual dimorphism in that trait, and heat tolerance may not acclimate as readily as other thermoregulatory traits^23,24^. Overall, our study suggests that, in some species, females may be at higher risk of (chronic, if not lethal) hyperthermia during heatwaves than males. These results are particularly concerning for population growth rates under climate change if female heightened susceptibility reduces breeding opportunity and reproductive success^36^, given females are often not tested in physiological studies, or sex is not reported (e.g.^19,39,41,51^).

Finally, our thermoregulatory values for free-living zebra finches are comparable to those for domestic or wild-derived captive populations of this model species, measured with a similar methodology^30,39^. There were however some differences, although they may have been driven by differences in acclimation conditions from those studies (e.g. mild constant T_a_ in captivity vs high and fluctuating in the wild). Indeed, at high T_a_, EHL/MHP was higher in wild individuals than individuals from captive populations (e.g. at T_a-chamb_ = 44 °C: mean = 2.1 vs 1.3 respectively^30^), possibly because of lower EWL in captive populations^39,51^. Accordingly, heat tolerance also appeared to be higher in wild zebra finches: 65% tolerated T_a-chamb_ = 44 °C for the whole 20-min stage duration versus only 27% in captive birds acclimated to 25 °C^30^. However, consistent with published values of zebra finch thermal limits^51^, at T_a-chamb_ = 46 °C, we had to terminate 45% of the trials on wild birds before 15 min (i.e. end of stage) to avoid the death of individuals showing severe signs of heat-stress. The heat tolerance limit of wild zebra finches thus may fall into the lower range of similarly-sized desert passerines^52^. However, EHL/MHP (mean = 2.6 at T_a-chamb_ = 46 °C) is above values described so far for this order^52,53^. This greater evaporative cooling capacity is partly attributable to the zebra finch being a drinking species^54^, but also potentially to the use of vocal panting (increasing heat dissipation through evaporative water loss^55^) and programming by prenatal “heat-calls”^56^. Indeed, incubating zebra finch parents produce heat-calls at high temperatures through an extreme form of panting, or “vocal panting”, which adaptively programs offspring development and phenotype for heat (e.g. higher reproductive success and heat tolerance^30,47,56^).

## Conclusion

To the best of our knowledge, this is the first study investigating the time course of acclimatisation of traits related to thermoregulation in the heat and sex differences in thermal acclimatisation in endotherms. Against the generally accepted view, we found that acclimatisation to heat occurred remarkably quickly—allowing individuals to track daily weather fluctuations, at least in a species adapted to highly variable weather. Our findings therefore strengthen the argument for considering phenotypic plasticity in climate change models to achieve realistic predictions. Importantly however, such phenotypic flexibility may not lessen the impact of heatwaves on populations, since heat tolerance did not respond, and only one of the sexes acclimatised. Our study highlights the need for further investigation of the acclimatisation capacities of species to heat, in order to understand the threat posed by climate change to biodiversity of endotherms.

## Materials and methods

### Study species and capture site

We examined heat tolerance and thermoregulation in free-living adult zebra finches (n = 31, 15 females and 16 males) during the austral summers of 2019–2020 and early 2021 (Fig. 1). To limit sources of inter-individual variation (and minimize impact on reproduction), we specifically targeted individuals moulting a few wing or tail feathers, since zebra finches tend to interrupt moulting during breeding^57^. The study took place at Wooltana station, South Australia (GPS: S 30.41324°, E 139.42035°) where daily maximum T_a_ ranged from 21 to 46 °C during data collection (Fig. 1). Birds were caught between 7:00 and 11:30 am, which corresponded to 1–5 h after sunrise. Since sunrise time varied slightly between field trips, we standardised the capture time (hereafter, “capture time”) by expressing it as the number of hours since sunrise (i.e. time at capture [7:00–11:30 am] minus sunrise time [6:08–6:57 am]). Birds were trapped at two capture sites 4 km apart, using feeder walk-in traps (filled with seed mix for a few days every 1 to 6 months) or in proximity of the feeder using mist nets. Birds were transported to a field laboratory (≤ 12 km away). We injected a temperature-sensitive passive integrated transponder (PIT) tag (Biomark, Boise, USA) subcutaneously into the bird’s flank. The PIT tags were covered by feathers and no noticeable moult in this area was observed. In small birds, including the zebra finch, subcutaneous PIT tags give similar values as those implanted intraperitoneally; subcutaneous PIT tags do not require surgery (superficial cut) and limit risk of fatal injuries^58^. The time since PIT tag injection also does not appear to affect measurement (Pessato and Mariette; unpublished data). We calibrated a subset of PIT tags in a water bath against a type-T thermocouple (BAT-12, Physitemp Instruments Inc., Clifton NJ, USA). Pit tags were accurate within 0.31 ± 0.06 °C across water temperature ranging from 40 to 46 °C.

All procedures were approved by Deakin University Animal Ethics Committee (B18-2017) and performed in accordance with Australian guidelines and regulations for the use of animals in research. This study was conducted in compliance with the ARRIVE guidelines (https://arriveguidelines.org).

### Experimental heat challenge protocol

Before respirometry measurements, birds were held in a cage with no food but ad libitum water. Based on the predicted mean retention time for food in a digestive tract for a 12 g bird (~ 50 min^59^) and to reduce interindividual variation, we applied a fasting time of 2 h when birds had seeds in their crop at capture or 1 h if the crop was empty. Just before starting each trial, birds were offered water by depositing drops on their bill, and were then weighed (mass ± 0.01, HT-120, A&D, Japan).

Heat exposure consisted of a stepped series of increasing air temperature (T_a-chamb_) in the metabolic chamber. The T_a-chamb_ was initially maintained at ~ 31 °C for 45 min (within the zebra finches’ thermoneutral zone^60^; but below the average maximum T_a_ over the two summers ~ 32.8 °C, Fig. 1), followed by 20-min stages at 35 °C, 40 °C, 42 °C, 44 °C and a 15-min stage at 46 °C. Trials were considered ‘complete’ when the individual remained in the chamber for 15 min at T_a-chamb_ = 46 °C. The trial was stopped early if the bird showed loss of balance, an abrupt drop in the water and CO_2_ traces, high body temperature (T_b_ > 45 °C) or prolonged escape behaviour^55,61^. Following the heat-exposure, birds were allowed to recover at thermoneutrality (T_a-chamb_ = 35 °C) for 10 min. Respirometry trials lasted on average 2h45 (range: 2h19–3h07). This stepped exposure protocol, involving brief periods at each T_a-chamb_ stage, yields similar results to exposure to each T_a-chamb_ for longer periods and is ethically (and practically) preferable^62^.

After measurements, birds were weighed, offered water and then transferred to a recovery cage for ~ 30 min with ad libitum water and finch seed mix, before release at their capture site. None of the birds died during the trials.

### Respirometry measurements during experimental heat challenge

We used an open flow-through respirometry system described in details by Pessato et al.^55^ to measure CO_2_ production and EWL. Briefly, all birds were placed individually in a metabolic chamber made of transparent plastic (1.5 L, 8 × 18 × 11.5 cm), containing a thermocouple to measure T_a-chamb_, a perch and a plastic mesh layer above a 5-mm layer of mineral oil. The chamber was placed into a dark temperature-controlled cabinet (Outermark, 99 × 51.5 × 48.5 cm) regulated by a temperature controller (ir33, Carel) connected to a thermocouple. We maintained very low humidity in the metabolic chamber (range: 0.1–0.9 kPa in excurrent air) by regulating incurrent airflow at flow rates of 2–3.5 L min^−1^ (depending on T_a-chamb_) with a mass flow controller (Alicat scientific Inc., USA, calibrated and accurate within 0.008 L min^−1^). Baseline and excurrent chamber air were sequentially subsampled and pulled by a pump (SS4 subsampler, Sable Systems) at ~ 240 mL min^−1^ through the H_2_O analyser (RH-300, Sable Systems) and CO_2_ analyser (CA-10, Sable Systems). Daily, the H_2_O analyser was zeroed using pure nitrogen (5.0, Coregas, Australia) and spanned with humidified air produced by a dew point generator (DG-4, Sable Systems). The CO_2_ analyser was zeroed and calibrated every 3 days using pure nitrogen and certified gas with a known CO_2_ concentration (1005 ppm, Coregas). Both analysers were connected to a computer interface (Expedata software and analog–digital converter UI2, Sable Systems). T_b_ was monitored every 10 s and recorded using a PIT tag reader (Biomark, Boise ID, USA); aberrant values (n = 3 out of 27,498 readings) were discarded.

Throughout the experiment, bird activity (i.e. movement) was monitored every 30 s for 5-s scans using infrared video cameras (mini CCD camera with IR, Signet). We scored activity following^30^ and restricted analyses to data to calm birds (i.e. sleeping, resting or stepping for small displacement) during measurement and in the 10 min prior.

### Respirometry data processing

In Expedata, for each T_a-chamb_ stage (31 °C, 35 °C, 40 °C, 42 °C, 44 °C, 46 °C), we selected the 1-min window with lowest and least variable CO_2_ and H_2_O values, after at least 29 min at the initial T_a-chamb_ stage (T_a-chamb_ = 31 °C), 9 min at T_a-chamb_ = 35 °C, 40 °C and 42 °C, and 6 min at T_a-chamb_ = 44 °C and 46 °C. We used the T_b_ in the 1-min window (accounting for 99% equilibrium time, ranging from 2 to 3.45 min depending on the flow rate^63^). We calculated (resting) metabolic rate (MR) and evaporative water loss (EWL) using equations 9.5 and 9.6 from^64^, and assuming a respiratory exchange ratio of 0.71 or 0.83, depending on crop content. After the trial, some birds (n = 5) still had seeds in their crop; therefore we assumed a respiratory exchange ratio of 0.71 (oxyjoule equivalent = 27.8 J ml^−1^ CO_2_) for birds with an empty crop at capture time (n = 8), and of 0.83 (oxyjoule equivalent = 24.9 J ml^−1^ CO_2_) for others (i.e. digesting seeds^65,66^). We also estimated evaporative cooling capacity, as evaporative heat loss over metabolic heat production (EHL/MHP) as EHL/MR. EWL was converted to evaporative heat loss (EHL in W) assuming a latent heat of vaporisation of 2.4 J mg^−1^ H_2_O^67^.

### Weather variables

We used weather data from Leigh Creek Airport meteorological station (station 017110, http://www.bom.gov.au) situated 95 km from Wooltana (the closest meteorological station to Wooltana (Arkaroola, 18 km away) had missing data; Pearson correlation between these stations: r = 0.91, p < 0.001). We used daily maximum T_a_ as we were interested in acclimatisation to heatwaves (correlation between maximum and minimum daily T_a_: r = 0.84, p < 0.001). We extracted the daily maximum T_a_ on the day of experiment (T_0day_), and the day before the experiment (T_−1day_). We also computed the average daily maximum air temperature over the preceding 3 days (T_−3days_), 1 week (T_−1week_) and 2 weeks (T_−2weeks_). To evaluate whether large deviations from conditions on the day prior to measurements affected acclimatisation, we also computed the difference in temperatures between T_0day_ and T_−1day_ (ΔT_0–1_) and T_−1day_ and T_−2days_ (ΔT_1–2_).

### Statistical analyses

All analyses were performed using R (v3.6.1) in RStudio (v1.1.1335). Of 31 birds used for measurements, we obtained thermoregulatory data for 29 birds, because of issues with humidity control in two trials. The PIT tag was not detected for one bird at T_a-chamb_ = 31 °C, so the sample size for T_b_ was n = 28 birds at T_a-chamb_ = 31 °C (and n = 29 at other T_a-chamb_ stages).

To investigate responses at mild and high T_a-chamb_, we considered separately T_a-chamb_ stages below or above the upper critical limit of thermoneutrality (T_uc_, i.e., inflection T_a-chamb_ in MR and EWL), identified at T_a-chamb_ = 38 °C in this data set, using broken line analyses (see supplementary information). In each dataset (below and above the T_uc_), we first defined the base model for each thermoregulatory variable (MR, EWL, EHL/MHP and T_b_), using linear mixed models (LMMs) with mass, T_a-chamb_ stage and/or capture time as predictors, and bird ID as a random factor, and selecting the model with the lowest Aikake Information Criterion corrected for small sample size (AICc^68^). Then, we identified the best weather predictors explaining variation in thermoregulatory variables, by adding one weather predictor at a time (Table S1) to the selected base model (Table S2), using LMMs and AICc. This approach allows testing which predictor, among a suite of correlated parameters (which thus cannot be considered jointly in a model), best explains the observed variation^69^. To build the model set for each thermoregulatory variable, we first (i) tested for the effects of temperatures at different timescales by adding to the base model either T_0day_, T_−1day_, T_−3days_, T_−1week_ or T_−2weeks_ as a predictor (model 2–6, Table S1). We then (ii) tested for the effect of sudden changes in temperatures by adding to the model with T_0day_ or T_−1day_, the deviation in temperature as either ΔT_0–1_ (i.e. T_0day_–T_−1day_) or ΔT_1–2_ (i.e. T_−1day_–T_−2days_) respectively (model 7–8, Table S1). Finally, (iii) to determine whether females and males differed in thermoregulatory performance, we fitted the same models as described above with sex as an additional predictor (model S1–S8, Table S1). We then used model averaging to test the significance of each parameter retained in the “top models”, within two AICc units of the best model, using the “model.avg” function from *MuMin* package and the conditional average method^69,70^. A predictor had a significant effect if its confidence interval excluded zero^71^. This method^70^ does not correct for multiple comparisons, which was not required in our case^72^.

To investigate the best predictors of heat tolerance, we fitted generalized linear models (GLMs) on two proxies of heat tolerance: the maximum T_a-chamb_ stage reached by an individual (T_a_-max = 46 °C or less) and whether or not the trial was completed (i.e. individual tolerated 15 min at T_a-chamb_ = 46 °C without showing sign of prolonged hyperthermia or activity). We used the model set and methods described above but without T_a-chamb_.

To verify the observed acclimatisation patterns were not driven by hidden seasonal effects, we repeated the above analyses on the data collected in February only (2020 and 2021, n = 19 birds with thermoregulatory data; Table S3).

Finally, to identify sex-specific predictor variables, we repeated the above procedure (model 0 to 8; Table S1) but considering males and females separately (and without sex as a predictor, Table S6). We used this split dataset approach rather than including interactions between sex and weather predictors because we were not aiming to test whether a particular time scale had opposite effects on the sexes, but instead, whether the same time scale was independently selected as best explaining variation in both of the sexes.

### Supplementary Information

Supplementary Information.

## Data Availability

Data are available on Mendeley: https://data.mendeley.com/datasets/kn6m7cg2p8/1.
