# Supplementary material for: Thermal acclimatisation to heatwave conditions is rapid but sex-specific in wild zebra finches
Source: Sci Rep. 2023 Oct 25;13:18297. doi: 10.1038/s41598-023-45291-0 (PMC10600105; doi:10.1038/s41598-023-45291-0)
Supplement: Supplementary file 1 — Supplementary Information. [file 41598_2023_45291_MOESM1_ESM.pdf]

Thermal acclimatisation to heatwave conditions is rapid but sex-specific in wild zebra finches

Supplementary information

Anaïs Pessato, Eve Udino, Andrew E. McKechnie, Andrew T. D. Bennett and Mylene M. Mariette*

Identification of the upper critical limit of thermoneutrality (T_{uc})

We investigated thermoregulatory variables (MR, EWL, EHL/MHP and T_b) as a function of increasing $T_{a\text{-}chamb}$ over the entire range. We determined if there was a significant inflection point for each of these variables, using the *Davies test* and *segmented* functions from the *segmented* R package (Muggeo, 2008).

We obtained almost identical values for inflection points for MR at $T_{a\text{-}chamb}$=37.6±1.2°C and EWL at 37.8±0.4°C. Inflection points for EHL/MHP and T_b were about one degree lower ($T_{a\text{-}chamb}$=36.8±0.7°C and 36.9±1°C, respectively, Figure S1).

<u>**Supplementary figures**</u>

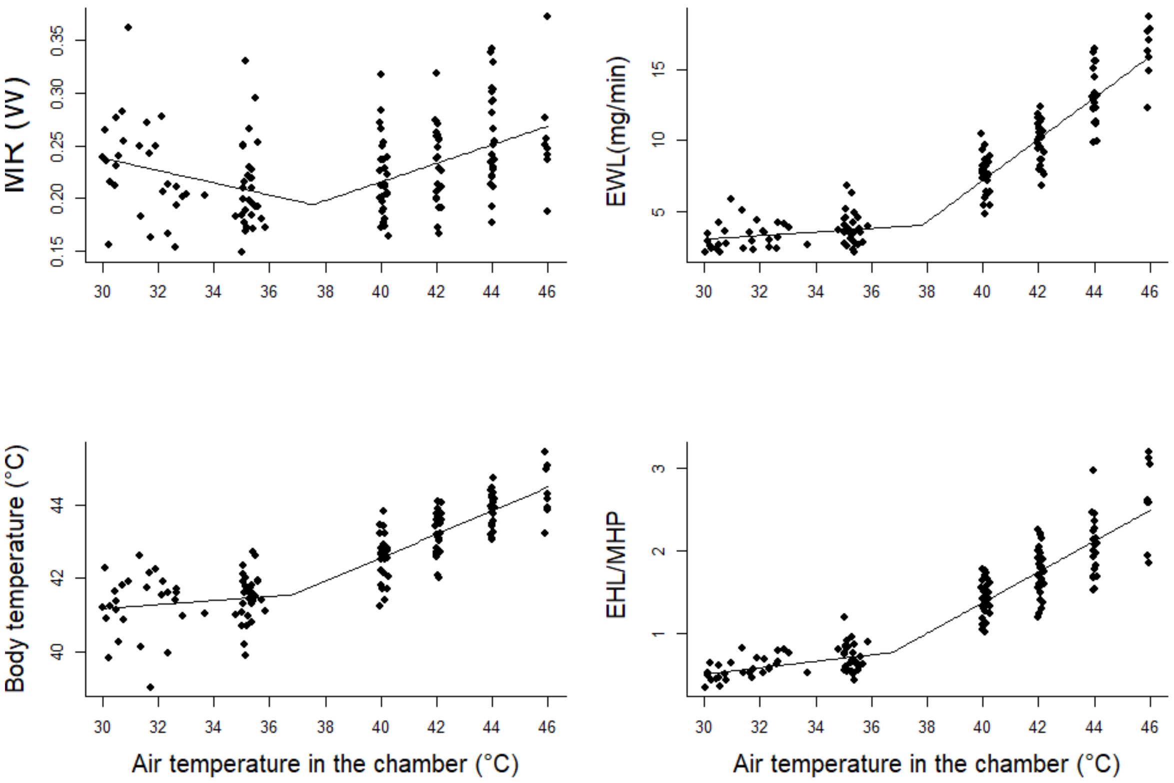

Figure S1: Broken line regressions as a function of air temperature in the chamber, above and below inflection points, for metabolic rate (MR), evaporative water loss (EWL), body temperature (T_b) and evaporative cooling capacity (EHL/MHP). *Regressions lines were estimated from the segmented function.*

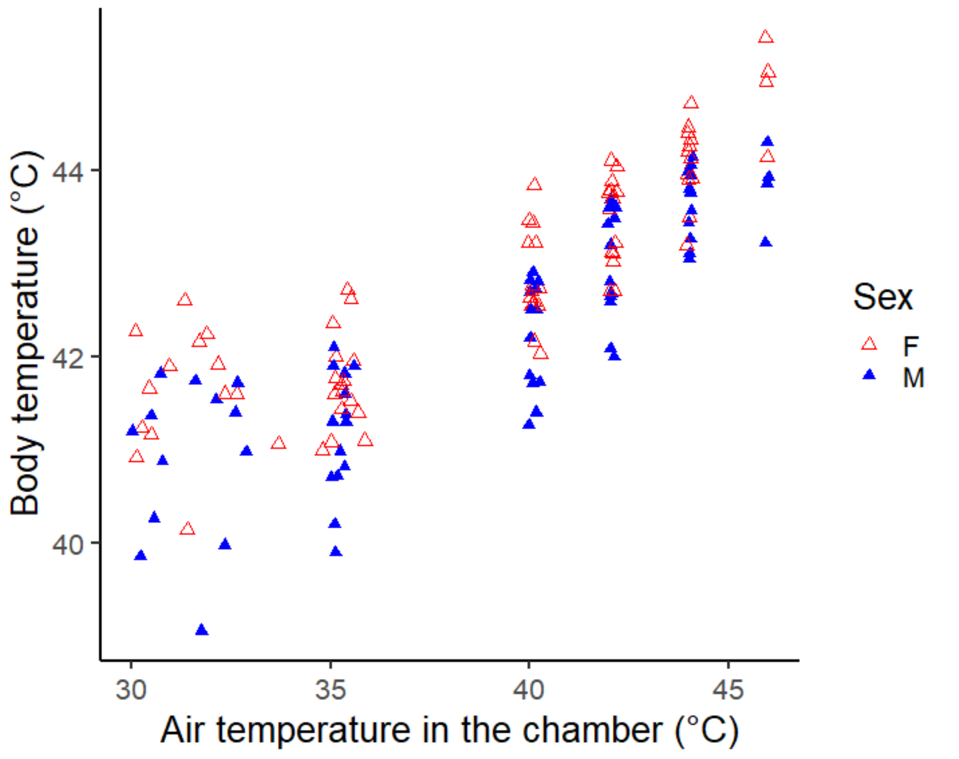

Figure S2: Body temperature T_b as a function of air temperature in the metabolic chamber. *Colours correspond to the sexes (open red triangles: females; filled blue triangles: males). Females had a higher T_b than males both below and above the T_{uc} (see main text).*

Supplementary tables

1. Method tables

**Model set**
0. Null model
Base model
1. Mass + $T_{a\text{-}chamb}$ + capture time
Effects of temperatures at different timescales
2. base model + T_{0day}
3. base model + T_{-1day}
4. base model + T_{-3days}
5. base model + T_{-1week}
6. base model + $T_{-2weeks}$
Effects of short term temperature deviation
7. base model + ΔT_{0-1} + T_{0day}
8. base model + ΔT_{1-2} + T_{-day1}
Effects of sex
S1-S8. Models 1 – 9 + sex

Table S1: Description of the model set used to identify the best predictors explaining variation in heat tolerance and thermal physiology.

2. Result tables

Whole dataset
Base model selection

Thermoregulatory variables									
		Below the T_{uc}				***Above the T_{uc}***			
	Best base model	*df*	*LL*	*AICc*	*w*	*df*	*LL*	*AICc*	*w*
**MR**	Null	3	103.7	-201.0	0.795	-	-	-	-
	$T_{a\text{-}chamb}$	-	-	-	-	4	172.2	-366.0	0.955
**EWL**	Mass	4	-79.2	167.3	0.380	-	-	-	-
	$T_{a\text{-}chamb}$ + mass	-	-	-	-	5	-136.9	284.5	0.653
**T_b**	$T_{a\text{-}chamb}$	4	-49.6	108.0	0.376	4	-40.2	88.8	0.639
**EHL/MHP**	Null	3	20.1	-33.8	0.524	-	-	-	-
	$T_{a\text{-}chamb}$	-	-	-	-	4	-0.142	8.8	0.735

Heat tolerance					
		df	*LL*	*AICc*	*w*
**$T_{a\text{-}max}$ = 46°C (yes/no)**	Capt. time	3	-19.76	46.4	0.363
**Trial completion**	Capt. time	3	-19.58	46.1	0.425

Table S2: Base model selection (before adding weather predictors): best base model explaining metabolic rate (MR), evaporative water loss (EWL), body temperature (T_b), evaporative cooling capacity (EHL/MHP) above and below the T_{uc} (n=29 birds), and heat tolerance variables on the full dataset (n=31). *The covariates mass, T_a and capture time (Capt. time) were considered.*

February-only data (no seasonal variation)
Base model selection

**Thermoregulatory variables**										
		Below the T_{uc}				***Above the T_{uc}***				
	Best base model	*df*	*LL*	*AICc*	*w*	*df*	*LL*	*AICc*	*w*
**MR**	Null	3	66.7	-126.7	0.848	-	-	-	-
	$T_{a\text{-chamb}}$	-	-	-	-	4	122.2	-325.6	0.965
**EWL**	$T_{a\text{-chamb}}$ + mass	5	-53.8	119.4	0.360	5	-92.9	196.9	0.605
**T_b**	$T_{a\text{-chamb}}$	4	-35.1	79.4	0.564	4	-22.1	52.9	0.715
**EHL/MHP**	$T_{a\text{-chamb}}$	4	12.1	-14.9	0.701	4	8.3	-7.9	0.739

**Heat tolerance**					
		df	*LL*	*AICc*	*w*
**$T_{a\text{-max}}$ = 46°C (yes/no)**	NULL	2	-14.01	32.7	0.602
**Trial completion**	NULL	2	-14.01	32.7	0.607

Table S3: Base model selection (before adding weather predictors): best base model explaining metabolic rate (MR), evaporative water loss (EWL), body temperature (T_b), evaporative cooling capacity (EHL/MHP) above and below the T_{uc} during February 2020 and 2021 (n=19 birds), and heat tolerance variables on the February dataset (n=21). *The covariates mass, T_a and capture time (Capt. time) were considered.*

February-only data
Weather predictor effects

Thermoregulatory variables

		Below the T_{uc}					***Above the T_{uc}***				
**Metabolic rate**	***(A)*** *Model*	*df*	*LL*	*AICc*	*ΔAICc*	*w*	*df*	*LL*	*AICc*	*ΔAICc*	*w*
	Null	3	66.7	-126.7	0	0.827					
	$+T_{-1day}$						5	123.8	-236.4	0	0.545
	Base						4	122.2	-235.6	0.7	0.376
	(B) *Predictors*	*Est.*		*SE*		*CI*	*Est.*		*SE*		*CI*
	Intercept	0.222		0.010		**0.20 ; 0.24**	0.238		0.007		**0.22; 0.25**
	$T_{a\text{-}chamb}$						0.039		0.005		**0.03; 0.05**
	T_{-1day}	-0.043		0.012		**-0.07 ; -0.02**	-0.042		0.012		**-0.07; -.0.02**
**Evaporative water loss**	***(A)*** *Model*	*df*	*LL*	*AICc*	*ΔAICc*	*w*	*df*	*LL*	*AICc*	*ΔAICc*	*w*
	Base	5	-53.8	119.4	0	0.182					
	$+T_{0day}$	6	-52.7	120.2	0.7	0.127					
	$+T_{-1week}$	6	-53.0	120.7	1.3	0.097					
	$+ T_{-1day}$	6	-53.0	120.7	1.3	0.095					
	$+T_{-2weeks}$	6	-53.1	120.9	1.4	0.089					
	$+ T_{-3days}$	6	-53.1	120.9	1.5	0.086					
	$+T_{-1week}$ + sex						7	-88.0	192.2	0	0.315
	(B) *Predictors*	*Est.*		*SE*		*CI*	*Est.*		*SE*		*CI*
	Intercept	3.449		0.190		**3.06 ; 3.84**	10.486		0.249		**10.03 ; 10.94**
	mass	1.178		0.405		**0.35; 2.00**	1.631		0.482		**0.75; 2.51**
	$T_{a\text{-}chamb}$	0.157		0.051		**0.05; 0.26**	5.406		0.262		**4.87; 5.91**
	T_{0day}	-0.591		0.409		-1.42 ; 0.24					
	T_{-1week}	-0.496		0.382		-1.27; 0.28					
	T_{-1day}	-0.498		0.409		-1.33; 0.33					
	$T_{-2weeks}$	0.469		0.389		-0.32; 1.26					
	T_{-3days}	-0.455		0.386		-1.24; 0.33					
	T_{-1week}						-1.190		0.503		**-2.11; -.027**
	Sex						1.250		0.505		**0.32; 2.18**
**Evaporative cooling capacity**	***(A)*** *Model*	*df*	*LL*	*AICc*	*ΔAICc*	*w*	*df*	*LL*	*AICc*	*ΔAICc*	*w*
	Base	4	12.1	-14.9	0	0.571	4	8.3	-7.9	1.9	0.103
	$+T_{0day}$						5	10.4	-9.7	0	0.263
	$+-T_{-1day}$						5	10.1	-9.0	0.7	0.183
	$+T_{-1day}$ + sex						6	11.3	-9.0	0.8	0.179
	(B) *Predictors*	*Est.*		*SE*		*CI*	*Est.*		*SE*		*CI*
	Intercept	0.6426		0.029		**0.57; 0.68**	1.764		0.056		**1.65; 1.88**
	$T_{a\text{-}chamb}$						0.636		0.036		**0.56; 0.71**
	T_{0day}						0.308		0.110		**0.09; 0.53**
	T_{-1day}						0.309		0.106		**0.10; 0.52**
	sex						0.245		0.103		**0.04; 0.45**

	(A) Model	df	LL	AICc	ΔAICc	w	df	LL	AICc	ΔAICc	w
**Body temperature**	+sex	5	-32.9	77.7	0	0.198	5	-20.6	52.3	0	0.218
	$+T_{0day}$ + sex	6	-31.7	78.2	0.5	0.154					
	$+T_{-2weeks}$ + sex	6	-32.3	79.3	1.6	0.090	6	-20.1	54.0	1.7	0.095
	$+T_{0day}$	5	-33.7	79.3	1.6	0.090					
	Base	4	-35.1	79.4	1.7	0.083	4	-22.1	52.9	0.6	0.164
	$+T_{-1week}$ + sex	6	-32.4	79.5	1.8	0.079					
	$+T_{-1day}$ + sex										
	***(B)** Predictors*	*Est.*		*SE*	*CI*		*Est.*	*SE*		*CI*	
	Intercept	41.318		0.160	**40.99; 41.64**		43.254	0.108		**43.04; 43.47**	
	$T_{a\text{-}chamb}$	0.078		0.19	**0.04; 0.12**		1.357	0.062		**1.23; 1.48**	
	Sex	-0.721		0.316	**-1.36; -.0.08**		-0.447	0.208		**-0.86; -0.03**	
	T_{0day}	-0.561		0.323	-1.22; 1.10						
	$T_{-2weeks}$	0.418		0.317	-0.23; 1.06		0.307	0.207		-0.11; 0.72	
	T_{-1week}	0.386		0.322	-0.27; 1.04						

Table S4: (A) Top model set (ΔAICc≤2 from best model), and (B) model-averaged estimates of predictors included in the top models, explaining variation in metabolic rate, evaporative water loss, evaporative cooling capacity and body temperature, below and above T_{uc} on the February dataset (n=19 birds). *A predictor has a significant effect (bold) if CI excludes 0. Female is the reference group for sex. Abbreviations: 'df': degree of freedom, 'LL': log-likelihood, 'ΔAICc': difference in AICc scores between the best model and the model being compared, 'w': model weight, 'Est.': parameter estimate, 'SE': standard error, 'CI': 95% confidence interval.*

February-only data
Weather predictor effects

Heat tolerance

	T_{a-max} = 46°C (yes/no)					Trial completion				
(A)										
Models	*df*	*LL*	*AICc*	*ΔAICc*	*w*	*df*	*LL*	*AICc*	*ΔAICc*	*w*
Null	**1**	**-13.4**	**28.9**	**0**	**0.207**	**1**	**-13.4**	**28.9**	**0**	**0.194**
$+\Delta T_{0-1} + T_{0day}$	**3**	**-11.2**	**29.7**	**0.8**	**0.140**					
$+T_{0day}$	**2**	**-12.9**	**30.4**	**1.5**	**0.100**	**2**	**-12.8**	**30.3**	**1.3**	**0.100**
$+T_{-1week}$	**2**	**-12.9**	**30.6**	**0.4**	**0.092**	**2**	**-12.4**	**29.5**	**0.6**	**0.145**
$+T_{-1day}$	**2**	**-13.1**	**30.8**	**1.9**	**0.081**	**2**	**-12.6**	**29.9**	**1.0**	**0.118**
$+T_{-3days}$	**2**	**-13.1**	**31.0**	**2.0**	**0.076**	**2**	**-13.0**	**30.6**	**1.7**	**0.084**

(B)						
Predictors	*Est.*	*SE*	*CI*	*Est.*	*SE*	*CI*
Intercept	0.766	0.510	-0.30; 1.83	-0.737	0.484	-1.75; 0.28
ΔT_{0-1}	-2.556	1.675	-6.07; 0.96			
T_{0day}	-0.768	1.105	-3.08; 1.55	1.048	1.027	-1.10; 3.20
T_{-1week}	0.873	0.969	-1.15; 2.90	1.416	1.107	-0.90; 3.73
T_{-1day}	0.715	0.943	-1.26; 2.69	1.352	1.250	-1.10; 3.20
T_{-3days}	-0.656	1.014	-2.78; 1.47	0.890	1.052	-1.31; 3.09

Table S5: (A) Top model set (ΔAICc≤2 from best model), and (B) model-averaged estimates of predictors included in the top models explaining variation in T_{a-max} (maximum $T_{a-chamb}$ reached = 46ºC or less) and trial completion (i.e. stayed 15 minutes at $T_{a-chamb}$=46°C) on the February dataset (n=21)*. A predictor has a significant effect (bold) if CI excludes 0. Abbreviations as in Table 1.*

Male and female datasets
Base model selection

Thermoregulatory variables									
		Below the T_{uc}				***Above the T_{uc}***			
	Best base model	*df*	*LL*	*AICc*	*w*	*df*	*LL*	*AICc*	*w*
MALES									
**MR**	Null	3	50.54	-94.1	0.969	-	-	-	-
	$T_{a\text{-}chamb}$	-	-	-	-	4	81.30	-153.5	0.866
**EWL**	Null	3	-34.42	75.8	0.428	-	-	-	-
	$T_{a\text{-}chamb}$ + mass	-	-	-	-	5	-61.51	134.7	0.478
**T_b**	Null	3	-25.21	57.5	0.477	-	-	-	-
	$T_{a\text{-}chamb}$	-	-	-	-	4	-14.99	39.1	0.648
**EHL/MHP**	Null	3	11.78	-16.6	0.789	-	-	-	-
	$T_{a\text{-}chamb}$ + mass	-	-	-	-	5	1.65	8.4	0.689
FEMALES									
**MR**	Null	3	50.46	-94.0	0.637	-	-	-	-
	$T_{a\text{-}chamb}$	-	-	-	-	4	85.45	-161.9	0.580
**EWL**	Mass	4	-44.00	97.6	0.368	-	-	-	-
	$T_{a\text{-}chamb}$ + mass	-	-	-	-	5	-71.71	155.0	0.634
**T_b**	Null	3	-22.29	51.5	0.628	-	-	-	-
	$T_{a\text{-}chamb}$	-	-	-	-	4	-22.17	53.3	0.433
**EHL/MHP**	Null	3	6.97	-7.0	0.811	-	-	-	-
	$T_{a\text{-}chamb}$	-	-	-	-	4	0.905	7.2	0.814

Table S6: Base model selection (before adding weather predictors): best base model explaining metabolic rate (MR), evaporative water loss (EWL), body temperature (T_b) and evaporative cooling capacity (EHL/MHP) above and below the T_{uc} when considering males (n=14) and females (n=15) separately. *The covariates mass, T_a and capture time (Capt. time) were considered.*

Male and female datasets

Weather predictor effects

Thermoregulatory variables

			MALES					FEMALES				
METABOLIC RATE	Below the T_{uc}	**(A)** Model	df	LL	AICc	ΔAICc	w	df	LL	AICc	ΔAICc	w
		Null	3	50.5	-94.1	0	0.829	3	50.5	-94.0	0.4	0.326
		$+T_{-1day}$	-	-	-	-	-	4	52.0	-94.4	0	0.400
		$+T_{-3days}$	-	-	-	-	-	4	51.3	-92.9	1.4	0.194
		**(B)** Predictors	Est.	SE	CI			Est.	SE	CI		
		Intercept	0.214	0.009	**0.19; 0.23**			0.223	0.010	**0.20 ; 0.24**		
		T_{-1day}	-	-	**-**			-0.060	0.017	**-0.10 ; -0.03**		
		T_{-3days}	-	-	-			-0.057	0.018	**-0.09 ; -0.02**		
	Above the T_{uc}	**(A)** Model	df	LL	AICc	ΔAICc	w	df	LL	AICc	ΔAICc	w
		Base	-	-	-	-	-	4	85.5	-161.9	0	0.338
		$+T_{-1day}$	5	86.5	-161.3	0	0.938	5	85.8	-160.0	1.9	0.133
		$+T_{-3days}$	-	-	-	-	-	5	86.4	-161.2	0.7	0.242
		$+T_{-1week}$	-	-	-	-	-	5	86.3	-161.0	0.9	0.212
		**(B)** Predictors	Est.	SE	CI			Est.	SE	CI		
		Intercept	0.231	0.005	**0.22 ; 0.24**			0.241	0.009	**0.22 ; 0.26**		
		$T_{a\text{-}chamb}$	0.035	0.007	**0.02 ; 0.05**			0.049	0.007	**0.04 ; 0.06**		
		T_{-1day}	-0.057	0.010	**-0.08 ; -0.04**			-0.047	0.016	**-0.08 ; -0.01**		
		T_{-3days}	-	-	**-**			-0.049	0.015	**-0.08 ; -0.02**		
		T_{-1week}	-	-	-			-0.048	0.015	**-0.08 ; 0.02**		
EVAPORATIVE WATER LOSS	Below the T_{uc}	**(A)** Model	df	LL	AICc	ΔAICc	w	df	LL	AICc	ΔAICc	w
		Null	3	-34.4	75.8	0.2	0.247	3	-46.0	99.0	1.4	0.129
		Base	-	-	-	-	-	4	-44.0	97.6	0	0.255
		$+T_{-2weeks}$	4	-33.0	75.7	0	0.268	-	-	-	-	-
		$+T_{-1week}$	4	-33.3	76.3	0.6	0.195	-	-	-	-	-
		$+T_{-1day}$	-	-	-	-	-	5	-43.0	98.6	1.0	0.156
		$+T_{-3days}$	-	-	-	-	-	5	-43.4	99.3	1.7	0.111
		**(B)** Predictors	Est.	SE	CI			Est.	SE	CI		
		Intercept	3.301	0.194	**2.90 ; 3.70**			3.619	0.240	**3.12 ; 4.11**		
		mass	-	-	-			0.852	0.508	-0.19 ; 1.90		
		T_{-1day}	-	-	-			-0.605	0.497	-1.63 ; 0.42		
		T_{-3days}	-	-	-			-0.432	0.541	-1.55 ; 0.68		
		T_{-1week}	0.604	0.392	-0.21 ; 1.41			-	-	-		
		$T_{-2weeks}$	0.678	0.382	-0.11 ; 1.47			-	-	-		
	Above the T_{uc}	**(A)** Model	df	LL	AICc	ΔAICc	w	df	LL	AICc	ΔAICc	w
		Base	-	-	-	-	-	5	-71.7	155.0	1.6	0.158
		$+T_{-3days}$	6	-58.8	132.0	0	0.351	-	-	-	-	-
		$+\Delta T_{1-2}+T_{-1day}$	7	-58.3	134.0	1.9	0.133	-	-	-	-	-
		$+T_{-1week}$	-	-	-	-	-	6	-69.6	153.4	0	0.350
		**(B)** Predictors	Est.	SE	CI			Est.	SE	CI		
		Intercept	10.829	0.227	**10.37 ; 11.29**			10.290	0.330	**9.62 ; 10.96**		
		$T_{a\text{-}chamb}$	5.776	0.282	**5.20 ; 6.35**			5.571	0.289	**4.99 ; 6.16**		
		mass	1.025	0.483	**0.05 ; 2.00**			1.741	0.694	**0.34 ; 3.14**		
		T_{-1day}	-1.411	0.620	**-2.67 ; -0.15**			-	-	-		
		ΔT_{1-2}	0.929	0.639	-0.37 ; 2.23			-	-	-		
		T_{-3days}	-1.113	0.470	**-2.07 ; -0.16**			-	-	-		

			Males					Females				
		T_{-1week}	-	-	-			-1.223	0.645	-2.53 ; 0.080		
EVAPORATIVE COOLING CAPACITY	Below the T_{uc}	*(A) Model*	*df*	*LL*	*AICc*	*ΔAICc*	*w*	*df*	*LL*	*AICc*	*ΔAICc*	*w*
		Null	3	11.8	-16.6	0.3	0.255	3	7.0	-7.0	0	0.781
		$+T_{-1day}$	4	13.3	-16.9	0	0.303	-	-	-	-	-
		$+T_{-2weeks}$	4	12.9	-16.0	0.9	0.191	-	-	-	-	-
		$+T_{0day}$	4	12.4	-15.0	1.9	0.119	-	-	-	-	-
		(B) Predictors	*Est.*	*SE*	*CI*			*Est.*	*SE*	*CI*		
		Intercept	0.626	0.026	**0.57 ; 0.68**			0.656	0.033	**0.59 ; 0.72**		
		T_{0day}	0.125	0.052	**0.02 ; 0.23**			-	-	-		
		T_{-1day}	0.143	0.050	**0.04 ; 0.25**			-	-	-		
		$T_{-2weeks}$	0.135	0.051	**0.03 ; 0.24**			-	-	-		
	Above the T_{uc}	*(A) Model*	*df*	*LL*	*AICc*	*ΔAICc*	*w*	*df*	*LL*	*AICc*	*ΔAICc*	*w*
		Base	5	1.6	8.4	1.5	0.192	4	0.9	7.2	0	0.577
		$+T_{0day}$	6	3.8	6.9	0	0.402	-	-	-	-	-
		$+T_{-1day}$	6	3.0	8.4	1.5	0.193	-	-	-	-	-
		(B) Predictors	*Est.*	*SE*	*CI*			*Est.*	*SE*	*CI*		
		Intercept	1.891	0.050	**1.79 ; 1.99**			1.689	0.66	**1.55 ; 1.82**		
		$T_{a-chamb}$	0.733	0.054	**0.62 ; 0.84**			0.587	0.051	**0.49 ; 0.69**		
		mass	0.259	0.111	**0.03 ; 0.48**			-	-	-		
		T_{0day}	0.302	0.101	**0.10 ; 0.51**			-	-	-		
		T_{-1day}	0.262	0.104	**0.05 ; 0.47**			-	-	-		
BODY TEMPERATURE	Below the T_{uc}	*(A) Model*	*df*	*LL*	*AICc*	*ΔAICc*	*w*	*df*	*LL*	*AICc*	*ΔAICc*	*w*
		Null	3	-25.2	57.5	0	0.311	-	-	-	-	-
		$+T_{0day}$	4	-24.1	58.0	0.5	0.239	4	-19.5	48.6	0	0.372
		$+T_{-1day}$	-	-	-	-	-	4	-20.1	49.8	1.1	0.214
		$+T_{-3days}$	-	-	-	-	-	4	-20.4	50.4	1.7	0.155
		(B) Predictors	*Est.*	*SE*	*CI*			*Est.*	*SE*	*CI*		
		Intercept	41.050	0.190	**40.66 ; 41.44**			41.670	0.116	**41.43 ; 41.91**		
		T_{0day}	-0.569	0.354	-1.30 ; 0.16			-0.646	0.229	**-1.12 ; -0.18**		
		T_{-1day}	-	-	-			-0.597	0.239	**-1.08 ; -0.11**		
		T_{-3days}	-	-	-			-0.565	0.245	**-1.07 ; -0.06**		
	Above the T_{uc}	*(A) Model*	*df*	*LL*	*AICc*	*ΔAICc*	*w*	*df*	*LL*	*AICc*	*ΔAICc*	*w*
		Base	-	-	-	-	-	4	-22.2	53.3	0	0.524
		$+T_{d0}$	5	-12.6	36.9	0	0.404	-	-	-	-	-
		(B) Predictors	*Est.*	*SE*	*CI*			*Est.*	*SE*	*CI*		
		Intercept	43.020	0.118	**42.78 ; 43.27**			43.547	0.111	**43.32 ; 43.77**		
		$T_{a-chamb}$	1.350	0.067	**1.21 ; 1.49**			1.333	0.088	**1.16 ; 1.51**		
		T_{0day}	-0.637	0.247	**-1.14 ; -0.14**			-	-	-		

Table S7: (A) Top model set (ΔAICc≤2 from best model), and (B) model-averaged estimates of predictors included in the top models explaining variation in MR, EWL, EHL/MHP and T_b below and above T_{uc}, in males (n=14, left) and females (n=15, right) considered separately. *A predictor has a significant effect if CI excludes 0 (bold). 'df' is degree of freedom, 'LL' is log-likelihood, 'ΔAICc' is the the difference in AICc score between the best model and the model being compared, 'w' corresponds to the model weight, 'Est.' to parameter estimate, 'SE' to standard error and 'CI' to the 95% confidence interval.*

Male and female datasets
Weather predictor effects

Heat tolerance

$T_{a\text{-}max} = 46°C$ (yes/no)

(A) Top models

	MALES					FEMALES				
Models	*df*	*LL*	*AICc*	*ΔAICc*	*w*	*df*	*LL*	*AICc*	*ΔAICc*	*w*
Null	1	-10.6	23.5	0.8	0.177	1	-9.5	21.4	0.4	0.173
Base	-	-	-	-	-	2	-8.0	21.0	0	0.216
$+T_{-1week}$	2	-8.9	22.6	0	0.268	-	-	-	-	-
$+T_{-1day}$	2	-9.4	23.8	1.2	0.150	-	-	-	-	-
$+T_{-2weeks}$	2	-9.5	24.0	1.3	0.137	3	-6.4	21.0	0.1	0.207
$+T_{-3days}$	2	-9.7	24.4	1.8	0.110	3	-7.2	22.5	1.5	0.101
$+ T_{0day}$	-	-	-	-	-	3	-7.2	22.7	1.7	0.092
$+\Delta T_{1\text{-}2}+T_{-1day}$						4	-5.5	22.9	2	0.081

(B) Model-averaged estimates of predictors included in the top models

	MALES			FEMALES		
Predictors	*Est.*	*SE*	*CI*	*Est.*	*SE*	*CI*
Intercept	0.574	0.561	-0.63 ; 1.78	0.879	0.701	-0.64 ; 2.40
mass	-	-	-	1.740	2.051	-2.71 ; 6.19
T_{0day}	-	-	-	-1.580	1.402	-4.63 ; 1.47
T_{-1day}	1.783	1.304	-1.01 ; 5.58	-	-	-
T_{-3days}	1.558	1.330	-1.29 ; 4.41	-2.169	2.053	-6.64 ; 2.30
T_{-1week}	2.407	1.593	-1.01 ; 5.82	-	-	-
$T_{-2weeks}$	1.766	1.350	-1.13 ; 4.66	-3.008	2.035	-7.44 ; 1.43
$\Delta T_{1\text{-}2}$	-	-	-	4.967	3.611	-2.98 ; 3;82

Trial completion

(A) Top models

	MALES					FEMALES				
Models	*df*	*LL*	*AICc*	*ΔAICc*	*w*	*df*	*LL*	*AICc*	*ΔAICc*	*w*
Null	-	-	-	-	-	1	-9.5	21.4	0	0.258
$+T_{-1week}$	2	-6.7	18.3	0	0.325	-	-	-	-	-
$+T_{-2weeks}$	2	-6.8	18.6	0.3	0.274	2	-8.5	22.1	0.7	0.186
$+T_{-1day}$	2	-7.3	19.6	1.3	0.166	2	-9.1	23.2	1.8	0.104
$+T_{-3days}$	-	-	-	-	-	2	-8.8	22.6	1.2	0.139
$+\Delta T_{0\text{-}1}+T_{0day}$	-	-	-	-	-	3	-7.6	23.3	1.9	0.098

(B) Model-averaged estimates of predictors included in the top models

	MALES			FEMALES		
Predictors	*Est.*	*SE*	*CI*	*Est.*	*SE*	*CI*
Intercept	-0.894	0.758	-2.51 ; 0.72	-0.799	0.624	-2.14 ; 0.54
T_{0day}	-	-	-	-0.483	1.402	-3.54 ; 2.57
$\Delta T_{0\text{-}1}$	-	-	-	3.468	2.274	-1.49 ; 8.42
T_{-1day}	4.455	2.764	-1.47 ; 10.38	-1.125	1.279	-3.89 ; 1.64
T_{-3days}	-	-	-	-1.578	1.467	-4.75 ; 1.59
T_{-1week}	5.702	3.219	-1.20 ; 12.61	-	-	-
$T_{-2weeks}$	3.763	1.801	-0.10; 7.63	-1.859	1.448	-4.99 ; 1.27

Table S8: (: (A) Top model set (ΔAICc≤2 from best model), and (B) model-averaged estimates of predictors included in the top models explaining variation in T_{a-max} (maximum $T_{a-chamb}$ reached = 46°C or less) and trial completion (i.e. stayed 15 minutes at $T_{a-chamb}$=46°C) in males (n=16, left) and females (n=15, right) considered separately. *A predictor has a significant effect if CI excludes 0. For both T_{a-max} and trial completion the base model was the null model. 'df' is degree of freedom, 'LL' is log-likelihood, 'ΔAICc' is the the difference in AICc score between the best model and the model being compared, 'w' corresponds to the model weight, 'Est.' corresponds to estimate, 'SE' to standard error and 'CI' is the 95% confidence interval.*

References

Muggeo, V. M. 2008. Segmented: an R package to fit regression models with broken-line relationships. *R news,* 8**,** 20-25.
